# Blast polytrauma with hemodynamic shock, hypothermia, hypoventilation and systemic inflammatory response: description of a new porcine model

**DOI:** 10.1007/s00068-020-01476-0

**Published:** 2020-08-30

**Authors:** Albin Dahlquist, Louise Elander Degerstedt, Erik von Oelreich, Andreas Brännström, Jenny Gustavsson, Ulf P. Arborelius, Mattias Günther

**Affiliations:** 1 Department of Clinical Science and Education, Section of Anaesthesiology and Intensive Care, Södersjukhuset, Karolinska Institutet, Sjukhusbacken 10, S1, 118 83 Stockholm, Sweden; 2 Department of Physiology and Pharmacology, Karolinska Institutet, Stockholm, Sweden; 3 Department of Neuroscience, Karolinska Institutet, Stockholm, Sweden

**Keywords:** Polytrauma, Blast injury, Haemorrhage, Porcine model

## Abstract

**Purpose:**

In the past decade blast injuries have become more prevalent. Blast trauma may cause extensive injuries requiring improved early resuscitation and prevention of haemorrhage. Randomized prospective trials are logistically and ethically challenging, and large animal models are important for further research efforts. Few severe blast trauma models have been described, which is why we aimed to establish a comprehensive polytrauma model in accordance with the criteria of the Berlin definition of polytrauma and with a survival time of > 2 h. Multiple blast injuries to the groin and abdomen were combined with hypoperfusion, respiratory and metabolic acidosis, hypoventilation, hypothermia and inflammatory response. The model was compared to lung contusion and haemorrhage.

**Methods:**

16 landrace swine (mean weight 60.5 kg) were randomized to “control” (*n* = 5), “chest trauma/hem” by lung contusion and class II haemorrhage (*n* = 5), and “blast polytrauma” caused by multiple blast injuries to the groin and abdomen, class II haemorrhage, lipopolysaccharide (LPS) infusion and hypothermia 32 °C (*n* = 6).

**Results:**

The blast polytrauma group had an Injury Severity Score of 57 which resulted in haemodynamic shock, hypothermia, respiratory and metabolic acidosis and inflammatory response. The chest trauma/hem group had an Injury Severity Score of 9 and less profound physiologic effects. Physiologic parameters presented a dose–response relationship corresponding to the trauma levels.

**Conclusion:**

A comprehensive blast polytrauma model fulfilling the Berlin polytrauma criteria, with a high trauma load and a survival time of > 2 h was established. A severe, but consistent, injury profile was accomplished enabling the addition of experimental interventions in future studies, particularly of immediate resuscitation efforts including whole blood administration, trauma packing and haemostasis.

## Introduction

Injury is a serious threat to public health, contributing to 1 in 10 mortalities and resulting in annual worldwide death of more than 5.8 million people [1, 2]. Blast injures account for an increasing number of deaths in both military and civilian life. In Iraq and Afghanistan, more than 71% of combat casualties in the US military were caused by explosions [3], and more than 1300 bombing incidents occur annually in the US [4]. In the past decade, sudden mass casualties due to bombings have become prevalent [2]. Blast events cause extensive injuries to multiple locations in the body leading to severe complications such as catastrophic haemorrhage and trauma-induced coagulopathy [2, 5]. Improved survival requires improved early and advanced resuscitation and prevention of bleeding [6, 7], but randomized prospective human trials are logistically and ethically challenging [8]. While further advancements require realistic animal models, few models of severe blast trauma have been described. Due to the complexity of blast injury it is unlikely that one model will be able to replicate all the relevant injuries and postinjury consequences [9]. Specifically, a model that encompasses a high trauma load is required, to complement established trauma models based on liver incisions or femur fractures [10–14]. It is known that coagulopathic complications increase with increased injury severity [15] and few models offer true ballistic trauma mechanisms [16, 17]. Therefore, we aimed to establish a comprehensive and ballistic polytrauma model, with a survival time of > 2 h, using multiple blast injuries to the groin and the abdomen, combined with hypoperfusion, respiratory and metabolic acidosis, hypoventilation, hypothermia and inflammatory response. The model was intended to fulfil the criteria of the Berlin definition of polytrauma [18]. ~ 60 kg swine were chosen based on the similarities to human anatomy and physiology [19]. Serving as a dose–response validation, the polytrauma model was compared to a model of chest trauma/hem caused by lung contusion [20]. We hypothesized that the model may be useful for studies of immediate resuscitation efforts, including whole blood administration, trauma packing and haemostasis, in civilian and military settings.

## Material and methods

**Fig. 1:**
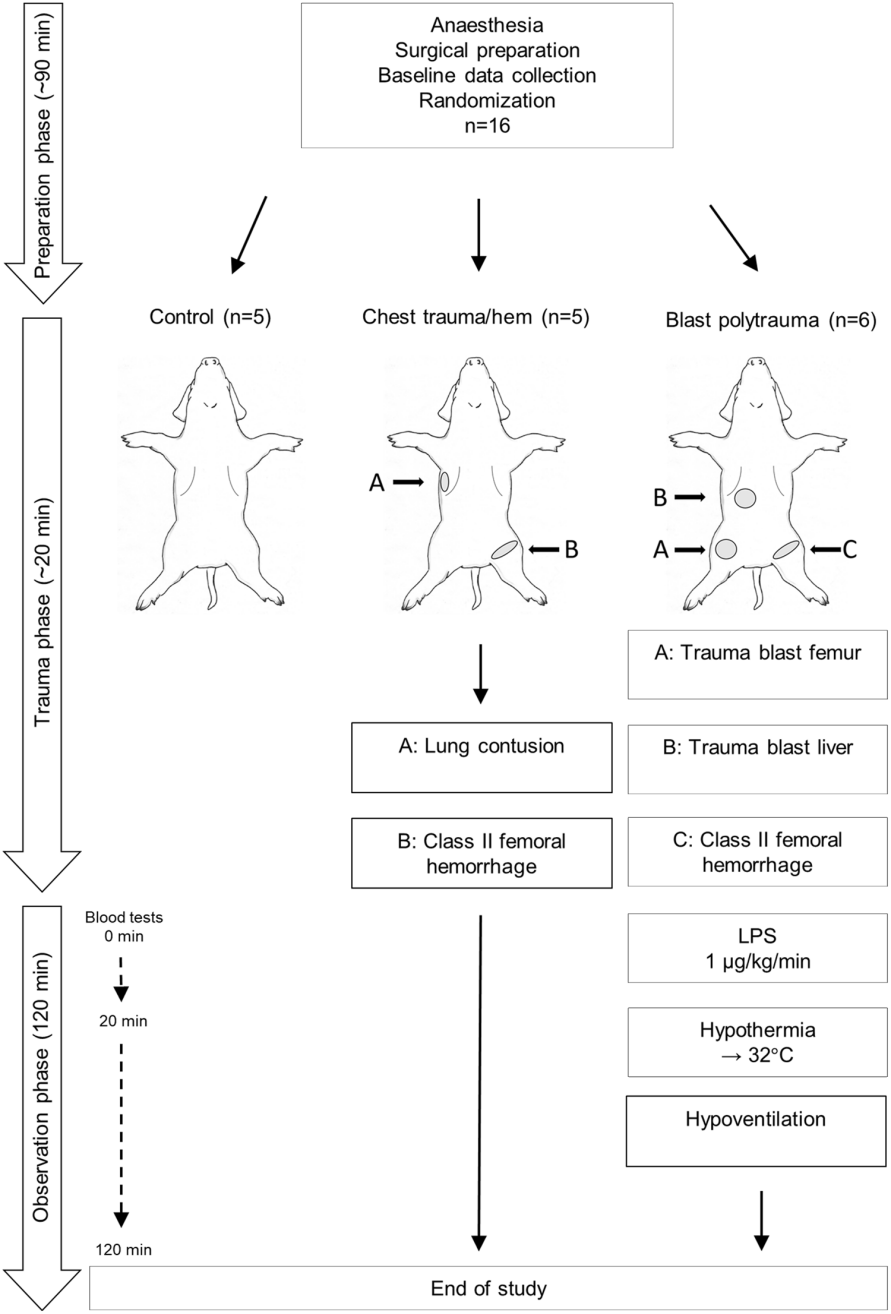
Chart of experimental setup and groups

All experiments were approved by the ethics committee in Linköping, Sweden (approval no: 1470). 16 landrace swine with a mean weight of 60.5 (range 56–64) kg were used. The experimental setup is described in Fig. 1.

### Preparation

Pre-medication consisted of 150 mg tiletamine/zolazepam (Zoletil 100 Vet) and 6 mg medetomidine (Domitor) after which anaesthesia was induced with fentanyl 2.5 µg/kg and pentobarbitalnatrium 6 mg/kg. Endotracheal intubation was performed with a custom-made Miller-type laryngoscope using a standard cuffed size 8 tube. Throughout the study, perioperative hypnosis was maintained with ketamine 25 mg/kg/h and midazolam 0.0485 mg/kg/h, and analgesia with fentanyl 3.5 μg/kg/h. The animals were ventilated with a Hamilton C2 (Hamilton Medical, Geneva, Switzerland) or a Servo 900C ventilator (Siemens-Elema, Solna, Sweden) using pressure control with initial settings PEEP 4, PIP 15 cm H_2_0, respiratory rate 15/min and FiO_2_ always remained at 21%. Settings were adjusted to achieve normoventilation before the onset of trauma (PaCO_2_ 4.9–5.7 kPa). Arterial blood gases [pH, PaCO_2_, PaO_2_, Na^+^, K^+^, Ca^++^, glucose, lactate, haematocrit (Hct), arterial blood saturation (SaO_2_) and base excess (BE)] were collected on baseline and then repeatedly throughout the experiment (GEM Premier 3000, Instrumentation Laboratories, Lexington, MA, USA). Whole blood haemoglobin analysis was measured with Hb 201^+^, HemoCue AB (Ängelholm, Sweden). A 7.5 F, 110 cm pulmonary artery catheter (Edwards Lifescience, Irvine, California USA) was cannulated in the right internal jugular vein via cut-down for monitoring of central venous pressure (CVP), cardiac output (CO), pulmonary artery pressure (PAP), mixed venous saturation (SvO_2_) and core temperature (Vigilance II-monitor, Edwards Lifescience). Perioperative monitoring by continuous electrocardiograms, and urine output were also performed. After an initial fluid bolus of 500 mL Ringer’s Acetate at induction of anaesthesia to correct for individual differences in preoperative fluid balance, the infusion rate was 3 mL/kg/h during anaesthesia. 100 mL boluses were given if mean arterial pressure (MAP) < 35 mmHg. No blood autotransfusion was performed. After preparation, animals were randomized to control (*n* = 5), chest trauma/hem (*n* = 5) or polytrauma (*n* = 6). Control animals were anesthetised for the duration of the experiment and were not exposed to any additional manipulation.

### Chest trauma/hem

**Fig. 2:**
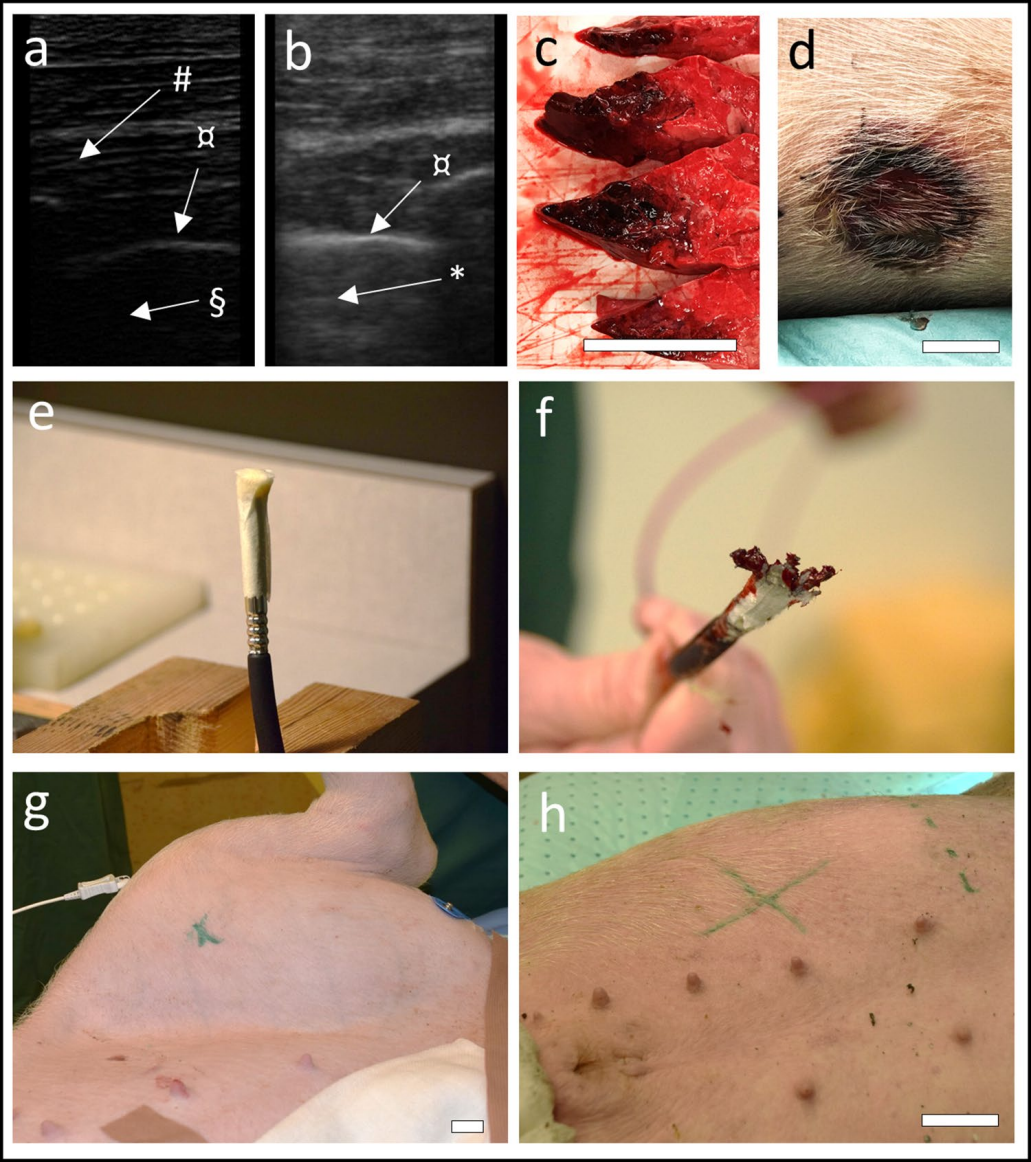
Photos of **a** ultrasound image of the lung with subcutaneous tissue (#), pleura (¤) and unaffected lung (§). **b** Ultrasound image of the lung with pleura (¤) and b-lines (*) disclosing intrapulmonary fluids. **c** Macro-histopathology specimens of the lung showing lung contusion. **d** Picture of thoracic skin lesion caused by impact of the projectile **e** detonator with 1 g of penthyl plastic explosive attached by tape. **f** Detonated explosive charge. **g** Placement of explosive charge above the femur, marked with a green cross. **h** Placement of explosive charge on the right abdomen above the liver, marked with a green cross. Size markers equal 5 cm

The animals were subjected to chest trauma by pulmonary contusion by a 59 g, 65 × 55 mm polyethylene projectile with mean velocity 82.3 (range 46–97) m/s, deployed 3.3 m from a custom-made cold air gun/compressed air gun, producing a 300-J energy-burst to the thorax. The projectile hit a fix point on the right dorsoanterior thorax (5 cm caudal and 2 cm ventral to the tip of the right scapula, 22–24 cm dorsally of the xiphoid process, right front leg in maximal abducted position) until pulmonary contusion was confirmed by B-lines and C-lines in ultra sound (Fig. 2a, b), which has sensitivity 94% and specificity 96% for lung contusion [21, 22] and haemoptysis was detected in the endotracheal tube (× 1 in 4 animals, × 3 in 1 animal). The consistency of the lung injury was confirmed in postmortem calculations of lung injury volume and skin lesion size (Fig. 2c, d) (data not shown). The chest trauma was followed by controlled haemorrhage from the femoral artery at a rate of approximately 100 mL/min until a class II haemorrhage was reached (25% of an estimated total blood volume of 65 mL/kg).

### Polytrauma

Swedish military grade plastic explosive (M/46) was utilized for the blast injuries. M/46 consists of 86% penthyl (pentaerytritoltetranitrat, C(CH_2_ONO_2_)_4_) and 14% mineral oil and has a detonation speed of 8400 m/s. 1 g of M/46 was taped to a non-electric initiation system capsule and 9 m of cord snapline SL-0, connected to a Nonel Dynostart ignition box (Dyno Nobel, Brisbane, Australia) (Fig. 2e, f). The explosive charge was taped to the skin and covered by ceramic plates from a military grade body armour. The animals were subjected to the following: (1) blast injury to the groin. The point of impact was above the mid-level of the femur (Fig. 2g). (2) Blast injury to the abdomen. The point of impact was 1 cm medially of the rib cage at level I6–I7 dx, corresponding to a level above the liver (Fig. 2h). The animals were then covered with flexible freezer packs to induce hypothermia. At completion of the blast injuries, lipopolysaccharide (LPS)-infusion (*Escherichia coli* O111:B4, Sigma-Aldrich, St.Louis, USA) was started (1 µg/kg/h), and the left femoral artery was exposed via cut-down technique and transected to induce uncontrolled haemorrhage. If MAP < 25 mmHg, the bleeding was temporarily stopped by compression. To ensure survivability during the observation period, vasopressor support by norepinephrine was given if MAP < 25 mmHg. The bleeding was terminated when a class II haemorrhage was reached (25% of an estimated total blood volume of 65 mL/kg). Following the blast injuries, hypoventilation causing hypercapnia was initiated by decreasing the minute ventilation. At completion of the experiments, the animals were euthanized by 40 mL pentobarbitalnatrium (Alfatal Vet 100 mg/mL) and post-mortem examinations were performed.

### Statistical analyses

Statistical analyses were performed using GraphPad Prism version 8.2.1 for Windows (GraphPad Software, La Jolla, Ca). For all temporal data sets, a mixed effects model with the Geisser–Greenhouse correction was used, comparing groups to the control group. For troponin T and myoglobin, paired *t* tests were used, comparing groups to the corresponding control group for the specific time. *p* < 0.05 was considered statistically significant. Error bars represent the standard error of the mean.

## Results

The chest trauma/hem resulted in Injury Severity Score 9 (head and neck: no injury, face: no injury, chest: serious, abdomen: no injury, extremity: no injury, external: no injury), and the polytrauma in Injury Severity Score 57 (head and neck: no injury, face: no injury, chest: severe, abdomen: critical, extremity: severe, external: severe).

**Fig. 3:**
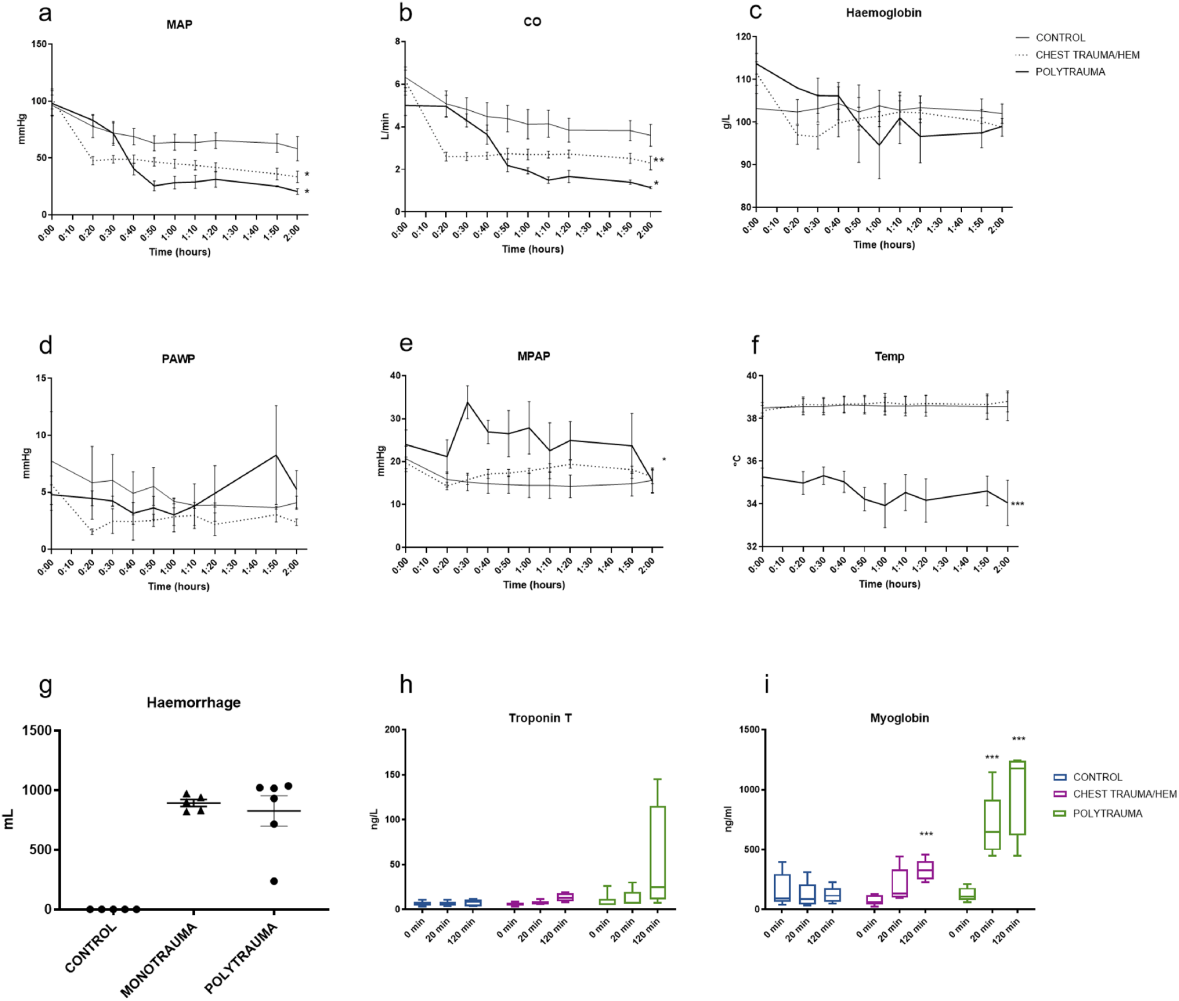
Circulatory and systemic effects. The polytrauma resulted in circulatory instability and hypothermia. **a** Mean artery pressure, MAP, **b** cardiac output, CO, **c** haemoglobin, **d** pulmonary artery wedge pressure, PAWP, **e** mean pulmonary artery pressure, MPAP, **f** temperature, **g** haemorrhage. **h** Troponin T, **i** myoglobin. *p < 0.05, **p < 0.01, ***p < 0.005

The panel in Fig. 3 describes circulatory consequences. MAP decreased in chest trauma/hem (*p* = 0.038, 95% CI of difference 1.458–38.14) and polytrauma (*p* = 0.023, 95% CI of difference 4.096–43.91) compared to controls (Fig. 3a). CO decreased in chest trauma/hem (*p* = 0.03, 95% CI of difference 0.1856–2.798) and polytrauma (*p* = 0.022, 95% CI of difference 0.2757–2.757) compared to controls (Fig. 3b). Haemoglobin remained unchanged compared to controls (Fig. 3c). Pulmonary artery wedge pressure (PAWP) remained unchanged compared to controls (Fig. 3d). MPAP increased in polytrauma (*p* = 0.015, 95% CI of difference − 16.44 to − 2.274) compared to controls (Fig. 3e). Temperature decreased in polytrauma (*p* = 0.001, 95% CI of difference 2.743–5.660) compared to controls (Fig. 3f). Mean haemorrhage was 892 mL in chest trauma/hem and 826 mL in polytrauma (Fig. 3g). Troponin T increased in polytrauma although not reaching statistical significance at 120 min compared to controls (Fig. 3h). Myoglobin increased in chest trauma/hem at 120 min (*p* = 0.015, 95% CI of difference − 200.3 to 61.99) and polytrauma at 20 min and 120 min (*p* = 0.0002, 95% CI of difference − 651.8 to − 300.3) (Fig. 3i).

**Fig. 4:**
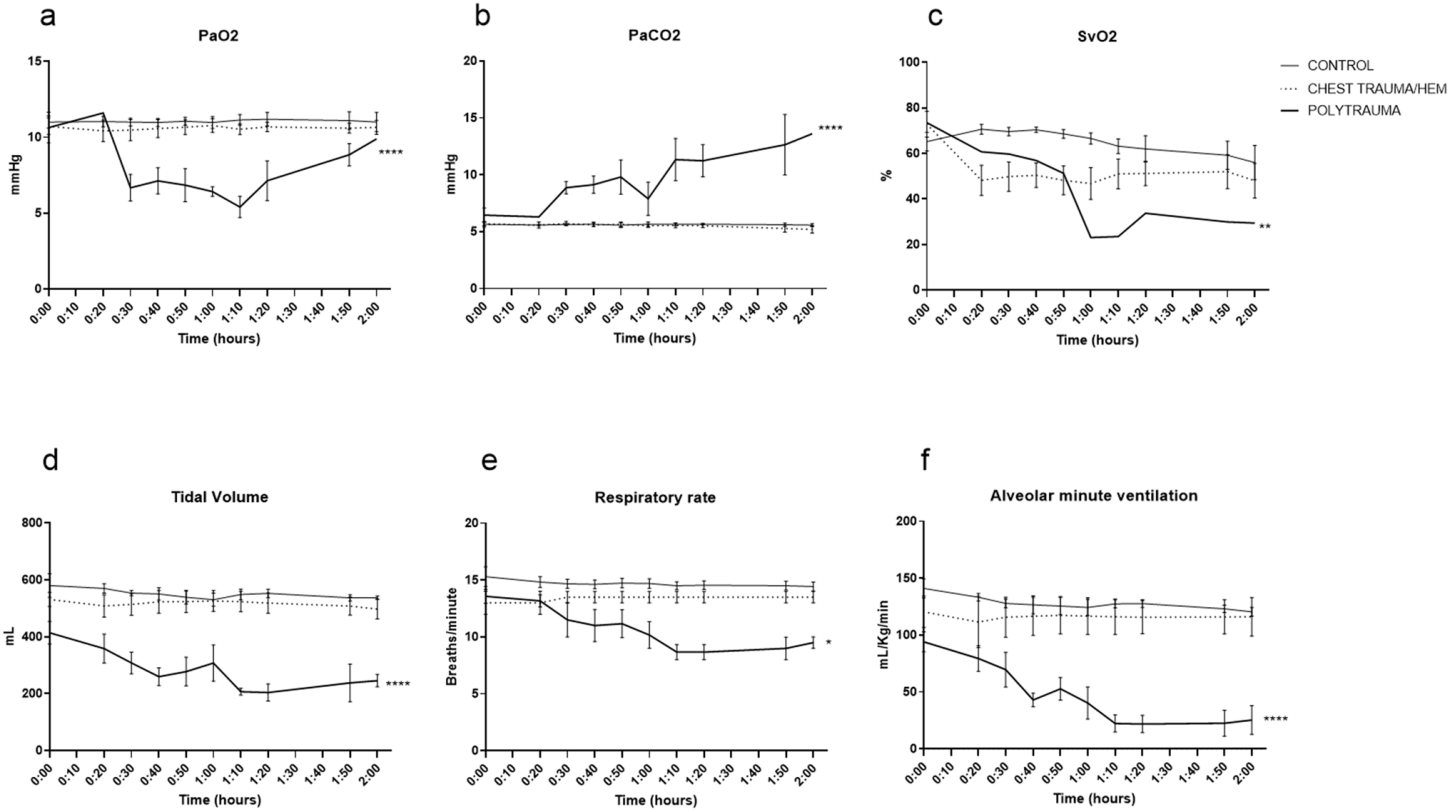
Respiratory effects. The polytrauma caused a severe hypercapnia and a transient hypoxia. **a** PaO_2_, **b** PaCO_2_, **c** SvO_2_, **d** tidal volume, **e** respiratory rate, **f** alveolar minute ventilation. *p < 0.05, **p < 0.01, ****p < 0.001

The panel in Fig. 4 describes ventilatory consequences. PaO_2_ decreased in polytrauma (*p* = 0.0062, 95% CI of difference 2.235–3.740) compared to controls. FiO_2_ was kept at 21% but oxygen tension did not fall below 5 mm Hg. (Fig. 4a). PaCO_2_ increased in polytrauma (*p* < 0.0001, 95% CI of difference − 5.143 to − 3.032) compared to controls (Fig. 4b). SvO_2_ decreased in polytrauma (*p* = 0.039, 95% CI of difference 1.663–40.25) compared to controls (Fig. 4c). Tidal volume decreased in polytrauma (*p* < 0.0001, 95% CI of difference 189.1–340.2) compared to controls (Fig. 4d). Respiratory rate decreased in polytrauma (*p*0.0058, 95% CI of difference 1.413–6.200) compared to controls (Fig. 4e). Alveolar minute ventilation decreased in polytrauma (*p* < 0.0001, 95% CI of difference56.78–99.24) compared to controls (Fig. 4e).

**Fig. 5:**
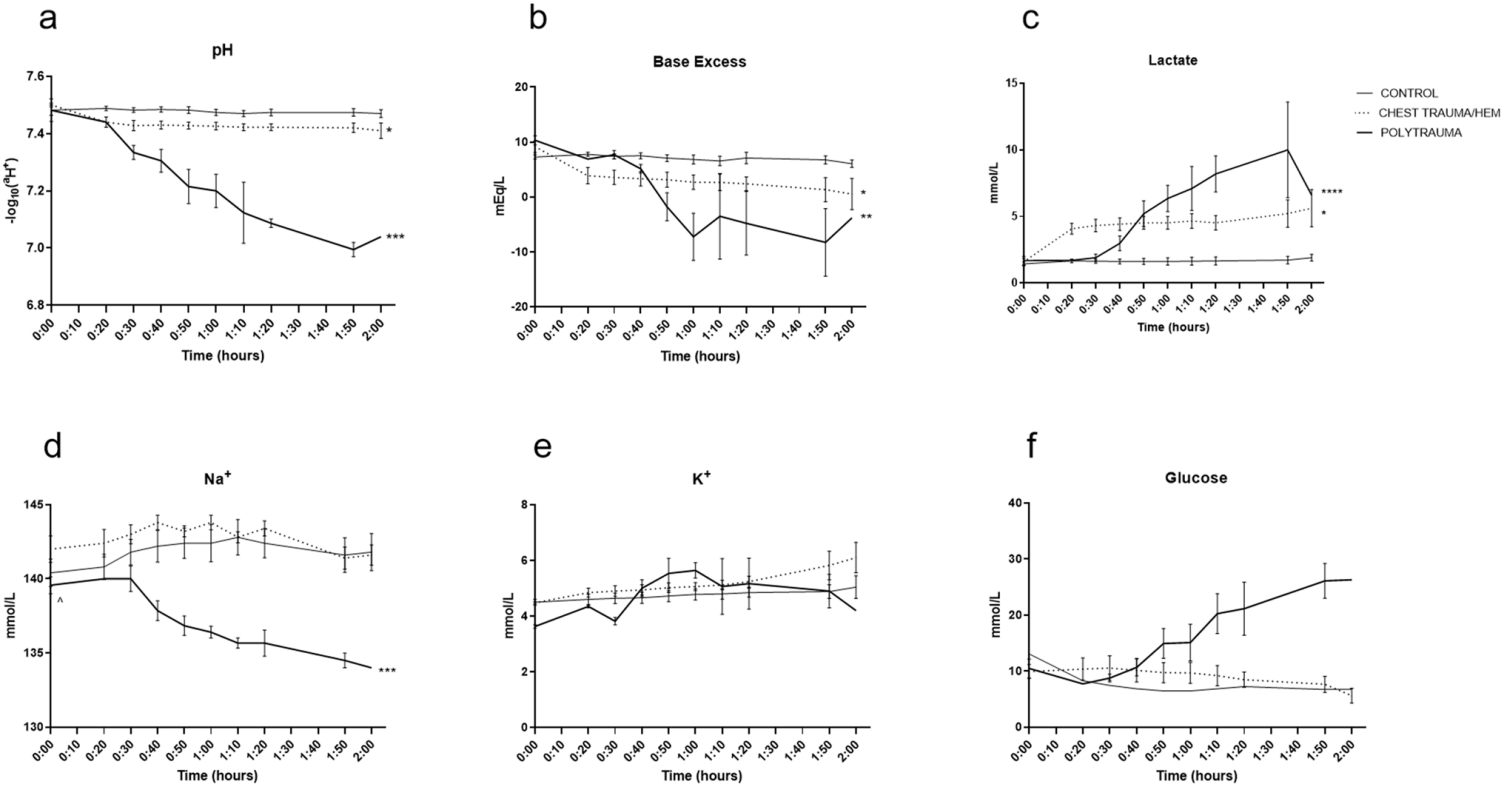
Metabolic effects. The polytrauma caused a combined severe respiratory and metabolic acidosis, hyponatremia and hyperglycaemia. **a** pH, **b** base excess, **c** lactate, **d** Na^+^, **e** K^+^, **f** glucose. *p < 0.05, **p < 0.01, ***p < 0.005, ****p < 0.001

The panel in Fig. 5 describes metabolic consequences. pH decreased in polytrauma (*p* < 0.0001, 95% CI of difference 0.1931–0.3412) and chest trauma/hem (*p* = 0.0179, 95% CI of difference 0.01016–0.08064) compared to controls (Fig. 5a). Base excess decreased in polytrauma (*p* = 0.0015, 95% CI of difference 4.227–12.32) and chest trauma/hem (*p* = 0.0479, 95% CI of difference 0.04467–7.475) compared to controls (Fig. 5b). Lactate increased in polytrauma (*p* = 0.0003, 95% CI of difference − 5.454 to − 2.381) and chest trauma/hem (*p* = 0.0025, 95% CI of difference − 4.132 to − 1.260) compared to controls (Fig. 5c). Sodium decreased in polytrauma (*p*0.0017, 95% CI of difference 2.076–6.646) compared to controls (Fig. 5d). Potassium remained stable compared to controls (Fig. 5e). Glucose increased in polytrauma although not reaching statistical significance compared to controls (Fig. 5f).

Mean norepinephrine administration was 0 in control, 0 in chest trauma/hem and 0.067 µg/kg/min in polytrauma. Mean total Ringer´s Acetate administration did not differ between groups and was 1517 mL in control (range 416 mL), 1780 mL in chest trauma/hem (range 892 mL) and 1230 mL in polytrauma (range 1359 mL).

## Discussion

In this study we describe a severe polytrauma model by multiple blast injuries and multiple exposure insults and a high trauma load of ISS 57. We aimed to create a platform for advanced prehospital and intrahospital resuscitation research, relevant for severe civilian or military trauma where trauma levels may be high [23]. A consistent and severe injury profile was attained and entailed severe tissue lacerations in the groin, a complex and multilevel femur fracture, complex liver lacerations, ventricle- and small intestine contusions and pulmonary contusions located caudally. The injuries were survivable during the observation period and did not result in malignant arrythmias or ventilatory complications.

The trauma model was designed to fulfil the criteria of the Berlin definition of polytrauma. The Berlin definition requires significant injuries of three or more points in two or more different anatomic Abbreviated Injury Scale (AIS) regions, in conjunction with one or more additional variable from five physiologic parameters (older age, unconsciousness, hypotension, acidosis, coagulopathy) [18]. The model was also designed to include all proposed causes of trauma-induced coagulopathy: severe trauma, hypoperfusion, respiratory and metabolic acidosis, hypoventilation, hypothermia and inflammatory response [24]. Potentially lethal complications by trauma-induced coagulopathy involve trauma in conjunction with acidosis, hypothermia and hypoperfusion [2, 25]. Our model complements existing porcine trauma models by increasing the injury load and combining hypoperfusion, hypoventilation and inflammatory response [26–32].

The polytrauma triggered a severe hypoperfusion due to hypotension and a decrease in cardiac output. The effects were less profound for mild chest trauma/hem and likely related to hypovolemia and systemic responses of the trauma. PAWP, a surrogate measure for cardiac preload, remained stable. This omitted heart failure as cause of hypotension [33]. Haemoglobin levels remained stable due to a deliberately conservative fluid resuscitation. To ensure survivability during the observation period, the polytrauma group was, therefore, stabilized by norepinephrine if needed. Vasopressor support is not standard treatment in trauma resuscitation and may not be available in a pre-hospital setting, why this comprises a limitation of the model. However, the model was designed to provide a near-fatal platform for specific and advanced resuscitation interventions, and we believe that intermittent vasopressor support in the absence of other resuscitation efforts was justified to assure survivability during the observation period. The quantity of vasopressor support given may also be useful as a marker of resuscitation efficacy in ensuing studies.

The metabolic derangements were dose–response related to the trauma and less profound in the mild trauma group. A similar dose–response was detected in tissue damage markers troponin and myoglobin. A deliberate hypoventilation was determined to be clinically relevant and led to a mixed respiratory and metabolic acidosis with a decreased pH, base excess and increased lactate. Interestingly, the polytrauma group displayed a hyponatremia while potassium remained unaffected. While hyponatremia is common in traumatic brain injuries [34], the association to polytrauma should be investigated in ensuing studies.

The polytrauma model was designed to include an inflammatory response during the 2-h observation period. Therefore, a low-level LPS infusion of 1 µg/kg/h was initiated after the trauma. LPS is a structural part of the outer membrane of Gram-negative bacteria, and one of the most effective stimulators of the immune system. LPS has been widely applied in swine in experimental models for bacterial infection with described infusion rates of 0.5–200 µg/kg/h [35]. However, LPS may be more artificial compared to methods of inflammation-induction such as faecal contamination of the peritoneal cavity. While future studies using this trauma model may consider omitting LPS-infusion for this reason, we show that LPS-infusion was possible after severe polytrauma and that advanced trauma models may utilize LPS to induce inflammatory response in models with hemodynamic instability. LPS allowed for a rapid, standardized and reproducible response during the observation time.

Another limitation of the model was the short observation time of 2 h, compared to described observation times of 48 h in milder polytrauma models [36]. However, when studying acute interventions after severe trauma, 2 h in the absence of resuscitation efforts are likely sufficient, and increased survival times may be expected when resuscitation is introduced in ensuing studies.

Human trials in severe trauma are difficult to design for logistical reasons and may be unethical. For this reason, prospective clinical data are scarce [29]. Animal models are thus invaluable when investigating pre-hospital trauma resuscitation and massive transfusion. Swine are considered a major animal species used in translational research and are increasingly used as the nonrodent species of choice. There is a large body of literature covering the normal anatomy and physiology of the swine [19]. Although anatomical variations exist compared to humans, the physiology of digestion, cardiovascular system and the lungs are remarkably alike. Differences include that of a spiral colon, increased amount of interlobular connective tissue in the liver and a hypercoagulative phenotype [19, 37]. While differences should be acknowledged and discussed when interpreting results from porcine trauma models, similarities between ~ 60 kg swine and humans make the animal model useful for advanced translational trauma research. There is increasing clinical and scientific interests in the use of early goal-directed coagulation therapies for haemostatic control in bleeding trauma patients [38], requiring relevant and new trauma models, particularly after blast injury [9]. We believe that this model will provide a platform for continued research, particularly of immediate resuscitation efforts after severe trauma.

## Conclusion

A comprehensive and severe blast polytrauma model fulfilling the Berlin polytrauma criteria, with a survival time of > 2 h, was established. A severe, but consistent, injury profile was accomplished enabling addition of experimental interventions in future studies.

## References

[1] GBD Causes of Death Collaborators (2018). Global, regional, and national age-sex-specific mortality for 282 causes of death in 195 countries and territories, 1980–2017: a systematic analysis for the Global Burden of Disease Study 2017. Lancet.

[2] Spahn DR, Bouillon B, Cerny V, Duranteau J, Filipescu D, Hunt BJ (2019). The European guideline on management of major bleeding and coagulopathy following trauma: fifth edition. Crit Care..

[3] Ritenour AE, Blackbourne LH, Kelly JF, McLaughlin DF, Pearse LA, Holcomb JB (2010). Incidence of primary blast injury in US military overseas contingency operations: a retrospective study. Ann Surg..

[4] Kapur GB, Hutson HR, Davis MA, Rice PL (2005). The United States twenty-year experience with bombing incidents: implications for terrorism preparedness and medical response. J Trauma..

[5] Eastridge BJ, Mabry RL, Seguin P, Cantrell J, Tops T, Uribe P (2012). Death on the battlefield (2001–2011): implications for the future of combat casualty care. J Trauma Acute Care Surg..

[6] Brannstrom A, Rocksen D, Hartman J, Nyman N, Gustavsson J, Arborelius UP (2018). Abdominal aortic and junctional tourniquet release after 240 minutes is survivable and associated with small intestine and liver ischemia after porcine class II hemorrhage. J Trauma Acute Care Surg..

[7] van der Burg BLSB, van Dongen T, Morrison JJ, Hedeman Joosten PPA, DuBose JJ, Horer TM (2018). A systematic review and meta-analysis of the use of resuscitative endovascular balloon occlusion of the aorta in the management of major exsanguination. Eur J Trauma Emerg Surg..

[8] Kauvar DS, Dubick MA, Martin MJ (2019). Large animal models of proximal aortic balloon occlusion in traumatic hemorrhage: review and identification of knowledge gaps relevant to expanded use. J Surg Res..

[9] Watts S, Kirkman E, Bieler D, Bjarnason S, Franke A, Gupta R (2019). Guidelines for using animal models in blast injury research. J R Army Med Corps..

[10] Hildebrand F, Weuster M, Mommsen P, Mohr J, Frohlich M, Witte I (2012). A combined trauma model of chest and abdominal trauma with hemorrhagic shock–description of a new porcine model. Shock..

[11] Dickneite G, Dorr B, Kaspereit F, Tanaka KA (2010). Prothrombin complex concentrate versus recombinant factor VIIa for reversal of hemodilutional coagulopathy in a porcine trauma model. J Trauma..

[12] Grottke O, Braunschweig T, Henzler D, Coburn M, Tolba R, Rossaint R (2010). Effects of different fibrinogen concentrations on blood loss and coagulation parameters in a pig model of coagulopathy with blunt liver injury. Crit Care..

[13] Cudjoe EK, Hassan ZH, Kang L, Reynolds PS, Fisher BJ, McCarter J (2019). Temporal map of the pig polytrauma plasma proteome with fluid resuscitation and intravenous Vitamin C treatment. J Thromb Haemost..

[14] Stormann P, Wagner N, Kohler K, Auner B, Simon TP, Pfeifer R (2019). Monotrauma is associated with enhanced remote inflammatory response and organ damage, while polytrauma intensifies both in porcine trauma model. Eur J Trauma Emerg Surg..

[15] Davenport RA, Brohi K (2016). Cause of trauma-induced coagulopathy. Curr Opin Anaesthesiol..

[16] Coakley TA, Devlin JJ, Kircher SS, Johnson AS (2012). Development of a ballistic model of combat groin injury. J Trauma Acute Care Surg..

[17] Hagemo JS, Jorgensen JJ, Ostrowski SR, Holtan A, Gundersen Y, Johansson PI (2013). Changes in fibrinogen availability and utilization in an animal model of traumatic coagulopathy. Scand J Trauma Resusc Emerg Med..

[18] Pape HC, Lefering R, Butcher N, Peitzman A, Leenen L, Marzi I (2014). The definition of polytrauma revisited: an international consensus process and proposal of the new 'Berlin definition'. J Trauma Acute Care Surg..

[19] Swindle MM, Makin A, Herron AJ, Clubb FJ, Frazier KS (2012). Swine as models in biomedical research and toxicology testing. Vet Pathol..

[20] Rocksen D, Gryth D, Druid H, Gustavsson J, Arborelius UP (2012). Pathophysiological effects and changes in potassium, ionised calcium, glucose and haemoglobin early after severe blunt chest trauma. Injury.

[21] Hyacinthe AC, Broux C, Francony G, Genty C, Bouzat P, Jacquot C (2012). Diagnostic accuracy of ultrasonography in the acute assessment of common thoracic lesions after trauma. Chest.

[22] Soldati G, Testa A, Silva FR, Carbone L, Portale G, Silveri NG (2006). Chest ultrasonography in lung contusion. Chest.

[23] Majde JA (2003). Animal models for hemorrhage and resuscitation research. J Trauma..

[24] Rossaint R, Bouillon B, Cerny V, Coats TJ, Duranteau J, Fernandez-Mondejar E (2016). The European guideline on management of major bleeding and coagulopathy following trauma. Crit Care..

[25] Kornblith LZ, Moore HB, Cohen MJ (2019). Trauma-induced coagulopathy: the past, present, and future. J Thromb Haemost..

[26] Watts S, Nordmann G, Brohi K, Midwinter M, Woolley T, Gwyther R (2015). Evaluation of prehospital blood products to attenuate acute coagulopathy of trauma in a model of severe injury and shock in anesthetized pigs. Shock..

[27] Mohr J, Ruchholtz S, Hildebrand F, Flohe S, Frink M, Witte I (2013). Induced hypothermia does not impair coagulation system in a swine multiple trauma model. J Trauma Acute Care Surg..

[28] Honickel M, Rieg A, Rossaint R, Braunschweig T, Spronk HM, ten Cate H (2011). Prothrombin complex concentrate reduces blood loss and enhances thrombin generation in a pig model with blunt liver injury under severe hypothermia. Thromb Haemost..

[29] Cho SD, Holcomb JB, Tieu BH, Englehart MS, Morris MS, Karahan ZA (2009). Reproducibility of an animal model simulating complex combat-related injury in a multiple-institution format. Shock..

[30] Reynolds PS, Fisher BJ, McCarter J, Sweeney C, Martin EJ, Middleton P (2018). Interventional vitamin C: a strategy for attenuation of coagulopathy and inflammation in a swine multiple injuries model. J Trauma Acute Care Surg..

[31] Spronk HM, Braunschweig T, Rossaint R, Wust DC, van Oerle R, Lauritzen B (2015). Recombinant factor VIIa reduces bleeding after blunt liver injury in a pig model of dilutional coagulopathy under severe hypothermia. PLoS ONE.

[32] Wong YC, Lai YY, Tan MH, Tan CS, Wu J, Zeng LZ (2015). Potential biomarker panel for predicting organ dysfunction and acute coagulopathy in a polytrauma porcine model. Shock..

[33] Vachiery JL, Adir Y, Barbera JA, Champion H, Coghlan JG, Cottin V (2013). Pulmonary hypertension due to left heart diseases. J Am Coll Cardiol..

[34] Rajagopal R, Swaminathan G, Nair S, Joseph M (2017). Hyponatremia in traumatic brain injury: a practical management protocol. World Neurosurg..

[35] Wyns H, Plessers E, De Backer P, Meyer E, Croubels S (2015). In vivo porcine lipopolysaccharide inflammation models to study immunomodulation of drugs. Vet Immunol Immunopathol..

[36] Eschbach D, Steinfeldt T, Hildebrand F, Frink M, Scholler K, Sassen M (2015). A porcine polytrauma model with two different degrees of hemorrhagic shock: outcome related to trauma within the first 48 h. Eur J Med Res..

[37] Velik-Salchner C, Schnurer C, Fries D, Mussigang PR, Moser PL, Streif W (2006). Normal values for thrombelastography (ROTEM) and selected coagulation parameters in porcine blood. Thromb Res.

[38] Caspers M, Maegele M, Frohlich M (2018). Current strategies for hemostatic control in acute trauma hemorrhage and trauma-induced coagulopathy. Expert Rev Hematol..

